# Gene expression analysis of human induced pluripotent stem cells cryopreserved by vitrification using StemCell Keep

**DOI:** 10.1016/j.bbrep.2021.101172

**Published:** 2021-11-15

**Authors:** Akemi Ota, Suong-Hyu Hyon, Shoichiro Sumi, Kazuaki Matsumura

**Affiliations:** a BioVerde Inc., Kyoto, Japan; b BMG Inc., Kyoto, Japan; c Department of Organ Reconstruction, Institute for Frontier Life and Medical Sciences, Kyoto University, Kyoto, Japan; d School of Materials Science, Japan Advanced Institute of Science and Technology, Nomi, Ishikawa, Japan

**Keywords:** Human induced pluripotent stem cell, Cryopreservation, Vitrification, DNA microarray, Gene enrichment analysis

## Abstract

In recent years, regenerative medicine research using human somatic and induced pluripotent stem cells has advanced considerably, promoting clinical applications. However, it is essential that these cells are cryopreserved safely and effectively. Most cryopreservation solution agents contain dimethyl sulfoxide (DMSO), which exhibits strong toxicity and can potentially promote cell differentiation. Hence, it is important to explore substitutes for DMSO in cryoprotectant solutions. One such alternative is StemCell Keep (SCK), a DMSO-free solution that has been reported to effectively cryopreserve human induced pluripotent stem cells (hiPS cells). To clarify the effect of cryopreservation agents on cells, DNA microarray analysis is useful, as it can identify a large number of gene expression differences in cryopreserved cells, as well as functional increases in gene groups. In this study, we performed gene expression analysis of SCK-cryopreserved hiPS cells using a DNA microarray gene chip. The hiPS cells vitrified with SCK or DMSO-based vitrification solutions were thawed and cultured on Matrigel under feeder-free conditions, and RNA was extracted for DNA microarray analysis. Genes obtained from DNA microarray data were classified by the keywords of Gene Ontology Biological Process Term, and their relationships were analyzed using DAVID or the GeneMANIA database.

SCK-cryopreserved hiPS cells expressed several anti-apoptotic genes, as well as genes related to cell adhesion or proliferation at levels that were nearly equivalent to those of non-frozen hiPS cells. Gene enrichment analysis with selected genes of SCK-cryopreserved hiPS cells whose expression differences were superior to those of DAP-cryopreserved showed strong interactions of negative regulation of apoptotic process, cell adhesion and positive regulation of cell proliferation in DAVID analysis. We demonstrated that SCK successfully maintained the key functions of hiPS cells, including anti-apoptosis, cell adhesion, and cell proliferation, during cryopreservation.

## Highlights

• Gene expression analysis of a DMSO-free solution SCK-cryopreserved hiPS was performed.
• SCK-cryopreserved hiPS cells maintained anti-apoptotic, adhesion, proliferation genes.
• SCK maintained the key functions of hiPS cells compared with current freezing solution.

## Abbreviations

SCK: StemCell Keep
hiPS cells: human induced pluripotent stem cells
hES cells: human embryonic stem cells
CPLL: carboxylated ε-poly –l-lysine
hPSC medium: human pluripotent stem cell medium
Oct3/4: Octamer-binding transcription factor 3/4
GO-BP: Biological Process Term in Gene Ontology
DAVID: the Database for Annotation, Visualization and Integrated Discovery
KEGG pathway: Kyoto Encyclopedia of Genes and Genomes pathway
AP: alkaline phosphatase
DAPI: 4,6-diamidino-2-phenylindole

## 1 Introduction

Human embryonic stem (hES) cells and human induced pluripotent stem (hiPS) cells are used clinically and in basic medical research, as they can be differentiated into somatic cells. The differentiated somatic cells can not only be transplanted into patients, but can also be used as clinical samples for assessing various treatment options. Thus, hES and hiPS cells allow the validation of therapeutic strategies without testing them on patients directly, and promote further research on the treatment of various diseases [[1], [2], [3]]. Given their importance, large-scale cryopreservation of hES and hiPS cells for prolonged periods is essential. Additionally, it is necessary to prevent any environmental damage in the cryopreserved hES or hiPS cells because of maintaining their high multiplicity and pluripotency [4]. Hence, the development of cryopreservation agents for stem cells is essential for their storage [5,6]. Vitrification is one of the low temperature preservation methods under development for the cryopreservation of large size cells, such as oocytes and embryos [7]. This method employs rapid freezing and has been utilized for large-scale cell-construct preservation in the field of tissue engineering [[8], [9], [10]].

We previously developed a potent cryoprotective solution, StemCell Keep (SCK), for the vitrification of stem cells, and demonstrated its effectiveness using hES and hiPS cells [11,12]. SCK is composed of carboxylated ε-poly-l-lysine (CPLL), a well-known cryoprotectant; ethylene glycol; and sucrose. We found that CPLL exhibited higher cryopreservation efficiency and lower cytotoxicity than dimethyl sulfoxide (DMSO), the industry standard for cryopreservation [13,14]. Although its mechanism of protection during freezing is not explicitly clear, CPLL may protect the cell membrane at low temperatures through the suppression of ice recrystallization [15,16], as well as by providing dehydration control to inhibit intracellular ice formation [17].

In previous studies, we observed that some stem cell-maker genes were highly expressed in hiPS cells cryopreserved in SCK compared with those exposed to a DMSO-based vitrification solution [12]; furthermore, DMSO was found to enhance unexpected differentiation in stem cells. We therefore examined a wide array of gene expression in hiPS cells cryopreserved with SCK [12,18,19]. The DMSO-based vitrification solution, DAP213 solution (DAP), composed of DMSO, acetamide, and propylene glycol, was first developed for the vitrification of mouse morulae and blastocysts [20]. DAP solution has also been used for the vitrification of hES cells and hiPS cells [21].

In this study, we investigated the gene expression profiles of SCK-cryopreserved hiPS cells by using a DNA microarray gene chip and compared them with the profiles of cells preserved with DAP.

## 2 Materials and methods

### 2.1 hiPS cell culture

The hiPS cells (253G1 strain, cell passage 9–12, RIKEN BioResourse Center (Tsukuba, Japan) were maintained on a feeder layer of mitomycin C-inactivated SNL 76/7 cells (SNL cells; DS Pharma Biomedical, Osaka, Japan) [[22], [23], [24]]. SNL cells were inoculated at a density of 1.6 × 10^4^ cells/cm^2^ on a 0.1% gelatin-coated 10 cm-plate and were cultured until 90% confluency. They were then mitotically inactivated by incubation with mitomycin C (10 μg/mL; Kyowa Hakko Kirin Co. Ltd., Tokyo, Japan) for 2–4 h, and were maintained on a 0.1% gelatin (Nacalai Tesque, Kyoto, Japan)-coated dish in high-glucose Dulbecco’s modified Eagle’s medium (Nacalai Tesque) containing 7% fetal bovine serum (FBS; MP Bio-Medicals, LLC Solon, OH, USA) and 1% penicillin–streptomycin (Nacalai Tesque) at 37 °C in a 5% CO_2_ incubator. The hiPS cells were maintained in human pluripotent stem cell (hPSC) medium (20% knockout serum replacement; Life Technologies, Carlsbad, CA, USA) containing 2 mM l-glutamine (Nacalai Tesque), 0.1 mM minimum essential medium with nonessential amino acids (Nacalai Tesque), 0.1 mM 2-mercaptoethanol (Life Technologies), and 5 ng/mL basic fibroblast growth factor (bFGF; Wako Pure Chemical, Osaka, Japan). hiPS cells were sub-cultured every 3–5 days using CTK buffer composed of 0.25% trypsin, 1 mg/ml collagenase (Life Technologies), 20% knockout serum replacement, and 1 mM CaCl_2_ (Nacalai Tesque) in phosphate buffer saline (PBS; Nacalai Tesque).

For feeder-free culture, hiPS cells were maintained at a density of 2 × 10^4^ cells/cm^2^ on 6-cm Matrigel-coated plates (BD Biosciences, Franklin Lakes, NJ, USA) in 5 mL of SNL-conditioned medium (supernatant of SNL cells cultured in hPSC medium at a density of 2 × 10^4^ cells/cm^2^ for one day, followed by further cultivation for two days before collection) containing 5 ng/mL bFGF at 37 °C in a 5% CO_2_ incubator.

Cells were then subcultured without the feeder layer, rinsed once with PBS, then dissociated with TrypLE Select (Life Technologies) to produce a single-cell suspension. The cells were then collected in a conical tube for centrifugation at 190*g* for 5 min, suspended in SNL-conditioned medium with 5 ng/mL bFGF, and plated on Matrigel-coated plates (BD Biosciences).

### 2.2 Vitrification and revival of hiPS cells

The vitrification solutions, SCK and DAP213 (DAP), were purchased from BioVerde (Kyoto, Japan) and Wako Pure Chemicals, respectively. For Vitrification, hiPS cells were cultured until 80% confluence, rinsed with PBS, and dissociated by treatment with CTK buffer. The detached hiPS cell colonies were gently pipetted to disperse clumps, collected in a conical tube (Thermo Fisher Scientific, Tokyo, Japan) for centrifugation at 190*g* for 5 min, and quickly suspended in 0.2 mL of ice-cold vitrification solution in a cryotube (Nunc). The tube was immediately immersed in liquid nitrogen and stored until required.

For the revival of vitrified hiPS cells, the cells were thawed by adding 1 mL of hPSC medium to the cryotube, and then transferred into a conical tube with 10 mL of hiPS cell medium for centrifugation at 190*g* for 5 min. The cells were suspended in 5 mL of SNL-conditioned medium with 5 ng/mL bFGF and 10 μM Y-27632 (Wako Pure Chemicals) onto Matrigel-coated 6-cm dishes and incubated for 24 h. After their revival, the SCK- and DAP-cryopreserved hiPS cells were cultured for one week. The medium was then replaced with SNL-conditioned medium containing bFGF only.

### 2.3 Alkaline phosphatase (AP) staining

A Leukocyte Alkaline Phosphatase Kit (Sigma-Aldrich, St. Louis, MO, USA) was used to assess the AP activity. Cultured hiPS cells were washed with PBS, fixed with 4% paraformaldehyde (Nacalai Tesque) for 10 min, then rinsed with distilled water. AP staining was carried according to the manufacturer’s instructions. After staining, the samples were washed with distilled water and air-dried.

### 2.4 Immunocytochemical analysis

The hiPS cells were cultured at the density of 1 × 10^5^ cells/cm^2^ in a 24-well plate overnight and were then rinsed with PBS and fixed as described in “Alkaline phosphatase(AP) staining”. The cells were permeabilized with Perm/Wash buffer I (BD Phosflow; BD Biosciences) for 15 min. After three washes with 2% FBS in PBS, the cells were incubated with diluted primary antibodies overnight at 4 °C. The primary antibodies used were Octamer-binding transcription factor 3/4 (Oct3/4; 1:200; Santa Cruz Biotechnology, Dallas, TX, USA) and Nanog (1:200; ReproCELL, Yokohama, Japan). The cells were washed, and secondary antibodies (Goat polyclonal Ab to rabbit IgG-FITC(1:300, Santa Cruz, CA, USA) and Goat F(ab) anti-mouse IgG-FITC (1:300, Santa Cruz, CA USA)) were added to the cells for 1 h in the dark. The samples were then washed three times with 2% FBS in PBS, and one drop of mounting medium containing 4,6-diamidino-2-phenylindole (Vector Laboratories, Burlingame, CA, USA) was added. The cells were observed under a fluorescence microscope (Keyence, Osaka, Japan). Negative controls were prepared using the same procedure, without primary antibody treatment.

### 2.5 DNA microarray experiments

The SCK- or DAP-cryopreserved hiPS cells were thawed and cultured on Matrigel under feeder-free conditions until 80% confluence. The cells, at an approximate concentration of 1 × 10^4^ cells/cm^2^ in a 6-cm dish, were rinsed once with PBS and dissociated using TrypLE Select (Life Technologies). Total RNA was extracted from hiPS cells using the RNeasy kit (Qiagen, Venlo, Netherlands) according to the manufacturer’s instructions. RNA samples from one 6-cm dish per sample were then handled by the Microarray Analysis Team at Kurabo Co. Biomedical Department (Osaka, Japan) for the DNA microarray experiments. The RNA concentration and purity were first assessed. The cDNA was synthesized from the RNA samples using the Whole Transcript Sense Target Labeling Assay Schematic (Affymetrix kit; Thermo Fisher Scientific) according to manufacturer instructions. The DNA microarray gene chip, GeneChip® 3' Expression Array Service(Affymetrix), was applied using the one-color method [25], and the signal data of each probe was calculated after normalization by the robust multi-array analysis (RMA) algorithm (Affymetrix Expression Console Software v.1.0 –User Guide 2013 130–132); the sample data were directly compared. The microarray results were provided on a DNA Microarray Viewer v.1.0 (Kurabo Co. Biomedical Department). The signal log ratio data showed the difference in expression between genes of interest.

The probes, gene names, chromosomal locations, NCBI Unigene IDs, and various database IDs have been provided at:

http://www.affymetrix.com/support/technical/manual/taf_manual.affx.

The genes that exhibited larger differences in gene expression were further analyzed with respect to their gene functions and connections through Gene Ontology and KEGG in DAVID, and with respect to higher gene networks in GeneMANIA.

### 2.6 Enrichment analysis of genes

Genes obtained from the DNA microarray data were classified based on the keywords of Biological Process Term in Gene Ontology (GO-BP) using the DNA Microarray Viewer software. We extracted genes with the GO-BP keywords, ‘apoptosis,’ ‘cell proliferation,’ ‘cell adhesion,’ and ‘stem cell,’ and whose expression difference between SCK-cryopreserved hiPS cells and non-frozen hiPS cells or between SCK-cryopreserved hiPS cells and DAP-cryopreserved hiPS cells was 1.4-fold or 1.5-fold [26]. Selected genes were explored with respect to the biological process, molecular function, cellular component annotations, and functional relationships or clustering using the Database for Annotation, Visualization, and Integrated Discovery (DAVID; http://david.abcc.ncifcrf.gov/) [27,28]. The gene list of interest was uploaded to ‘DAVID Functional Annotation Bioinformatics Microarray Analysis’, and were analyzed by the ‘Functional Annotation Tool’, mainly using the categories from Gene Ontology and the Kyoto Encyclopedia of Genes and Genomes (KEGG) pathway [29,30]. The ‘Probe set ID’ of selected genes obtained from the DNA microarray data were first translated into Entrez Gene IDs using the gene conversion tool, and then introduced into the gene functional annotation tool. Finally, the gene category annotated based on the DAVID score enrichment p-value (*p* < 0.05) was considered.

GeneMANIA was used to identify genes related to sets of selected genes underlying specific functional themes, as identified by DNA microarray data, and the Gene Symbol of each was uploaded for analysis. The GeneMANIA algorithm comprised a linear-regression-based algorithm for calculating single, composite, functional association networks from multiple networks derived from different proteomic or genomic data sources, and for the prediction of gene function [31,32].

### 2.7 Statistical analysis

The counts of AP + colonies have been presented as mean ± standard deviation. Statistical analyses were performed using Excel Statistics (SSRI Co. Ltd., Tokyo, Japan). The student’s *t*-test was used for analyzing data when two groups were compared. Statistical significance was set at *p* < 0.05. In order to compare the fluctuations in gene expression in the DNA microarray data, the *p*-value of the *t*-test, Benjamini-Hochberg method, and false discovery ratio in DAVID analysis were considered.

## 3 Results

### 3.1 Vitrification of hiPS cells

**Fig. 1:**
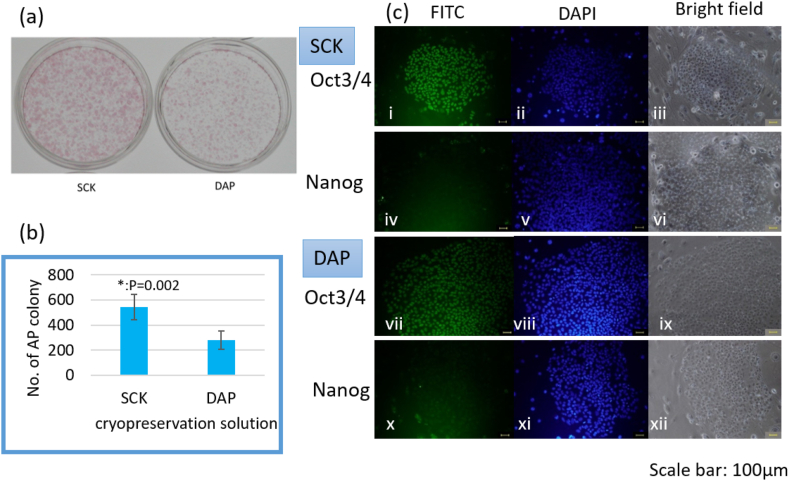
Characterization of StemCell Keep (SCK)-cryopreserved human induced pluripotent stem (hiPS) cells. (a) Representative images of alkaline phosphatase (AP) + staining of SCK- or DAP213 (DAP)-cryopreserved hiPS cells. (b) Number of AP + colonies that generated from SCK- or DAP-cryopreserved hiPS cells. *p = 0.002 (c) Representative images of immunofluorescence staining of SCK- or DAP-cryopreserved hiPS cells. Oct 3/4-FITC staining (green), i and vii; Nanog-FITC staining (green), iv and x; DAPI, 4,6- diamidino-2-phenylindole, staining (blue), ii, v, viii and xi; bright-field images, iii, vi, ix and xii. Scale bar: 100 μm. . (For interpretation of the references to color in this figure legend, the reader is referred to the Web version of this article.)

Proliferation and multipotency of SCK-cryopreserved hiPS cells for 1 week was first assessed. The number of AP + colonies generated by the SCK-cryopreserved hiPS cells (546 ± 101) was significantly higher than that of DAP-cryopreserved hiPS cells (282 ± 74; *p =* 0.002, Fig. 1a and b). Furthermore, the pluripotent markers Oct3/4 and Nanog [33], were found to be expressed in both SCK- and DAP-cryopreserved hiPS cells by immunocytochemical staining (Fig. 1c). Thus, the SCK-cryopreserved hiPS cells maintained their pluripotency and multipotency even after revival.

### 3.2 Enrichment analysis of ‘apoptosis’ in SCK-cryopreserved hiPS cells

**Fig. 2:**
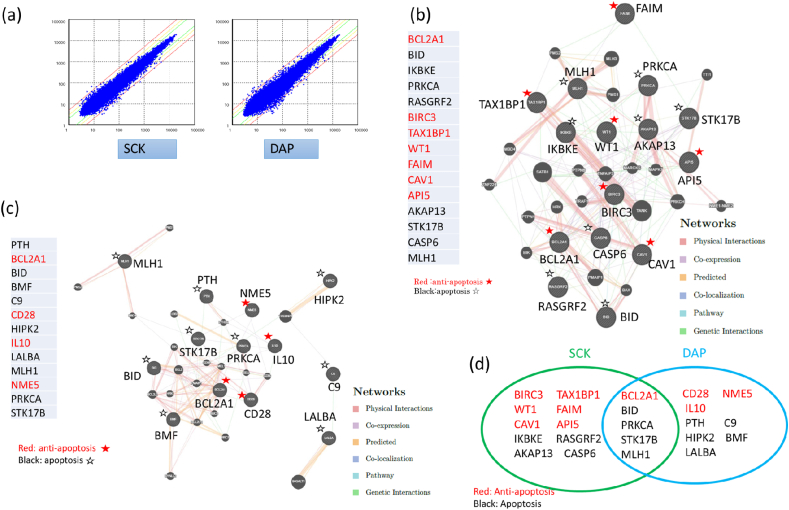
Annotation analysis profiles of SCK- and DAP- cryopreserved hiPS cells (I). (a) Dot plots of SCK- and DAP- cryopreserved hiPS cells in DNA microarray data. In dot plots, red or green line upper diagonal shows 2 or 1 of signal log ratio, respectively, and those of lower diagonal shows -2 or -1 of signal log ratio, respectively. (b) GeneMANIA profile of genes of SCK-cryopreserved hiPS cells classified under ‘apoptosis’ with a difference in expression of more than 1.5-fold compared with non-frozen hiPS cells. Red font indicates anti-apoptotic genes, and black indicates apoptotic genes. (c) GeneMANIA analysis of genes in DAP-cryopreserved hiPS cells classified under ‘apoptosis’ with a difference in expression of more than 1.5-fold compared with the non-frozen hiPS cells. Red font indicates anti-apoptotic genes, and black indicates apoptotic genes. (d) Correlation diagram of genes classified under ‘apoptosis’ in Biological Process Term of Gene Ontology with a difference in expression of more than 1.5-fold between SCK- or DAP- cryopreserved cells and non-frozen hiPS cells. Red font indicates anti-apoptotic genes, and black indicates apoptotic genes. . (For interpretation of the references to color in this figure legend, the reader is referred to the Web version of this article.)

Next, we investigated differences between gene expression profiles of SCK- and DAP-cryopreserved hiPS cells and non-frozen hiPS cells, which had been maintained in normal culture conditions for at least three or four passages after reviving. The dot plots of DNA microarray data of SCK and DAP are shown in Fig. 2 and Fig. S1. The GeneChip Gene 2.0ST array probe set (Probe number: 56317) was used. We analyzed the data using DNA Microarray Viewer software, and selected four keywords—apoptosis, cell adhesion, cell proliferation, and stem cell—categorized in GO-BP, all of which were important factors for hiPS cell culture and may help further their use in research.

Notably, many hiPS cells do not survive post-revival due to apoptosis [34]. Hence, we extracted apoptotic genes and assessed the difference in the expression of these genes during the freeze-thaw period. For the remaining three keywords, cell adhesion, cell proliferation, and stem cell, we compared the cryopreservation effects of SCK and DAP on hiPS cells. The genes extracted by each keyword were subjected to an enrichment analysis using DAVID, which was converted from the Probe Set IDs of DNA microarray data into Entrez Gene IDs, and categorized into Gene Ontology or KEGG pathway. Altitude gene networks of each keyword were further extracted using GeneMANIA, and their gene networks were investigated.

Table 1 shows the annotation analysis scheme of DNA microarray data (the data obtained from the microarray experiments were selected from four groups of the GO-BP by DNA Microarray Viewer), as well as the difference in gene expression between the SCK- or DAP-cryopreserved hiPS cells and non-frozen hiPS cells, or between the SCK- cryopreserved and DAP-cryopreserved hiPS cells. We extracted 15 genes using the DNA Microarray Viewer that were differentially expressed by over 1.5-fold between the SCK-cryopreserved and non-frozen hiPS cells. Among these genes, seven were categorized as anti-apoptotic (*BIRC3, TAX1BP1*, *BCL2A1, WT1, FAIM, CAV1* and *API5*), while the remaining eight were categorized as apoptotic (*BID, PRKCA, IKBKE, RASGRF2, STK17B, AKAP13, CASP6*, and *MLH1*), though *PRKCA* exhibited both apoptotic and anti-apoptotic functions in glial cells (Table 2a).

**Table 1:** Annotation analysis scheme of DNA microarray data.

1) Data Grouping with the category of Biological Process Term in Gene Ontology		
No.	Group	
1	Apoptosis	
2	Cell adhesion	
3	Cell proliferation	
4	Stem cell	
2) Data classification		
No.	Classification	
1	Gene expression difference between SCK- or DAP-cryopreserved hiPS cells and non-frozen hiPS cells	
2	Gene expression difference between SCK-cryopreserved hiPS cells and DAP-cryopreserved hiPS cells	
3) Data analysis tool		
No.	Software	Contents
1	DNA Microarray Viewer	Signal log ratio
		Probe set ID
2	Gene Ontology	Biological Process Term
3	DAVID	Entrez Gene ID
		Net work category
		KEGG
4	GeneMANIA	Gene network

**Table 2a:** Genes of SCK-cryopreserved hiPS cells classified under ‘apoptosis’ with a difference in expression of more than 1.5-fold compared with the non-frozen hiPS cells.

No.	Gene Symbol	Probe Set ID	control2_Signal	SCK_Signal	SCK_Signal Log Ratio	GO Biological Process Term	Pathway Name
1	BCL2A1	16812344	40.4	77.5	0.94	apoptotic process//anti-apoptosis	Apoptosis_KEGG
2	BID	16932008	309.5	587.0	0.92	induction of apoptosis via death domain receptors//glial cell apoptotic process//positive regulation of apoptotic process//neuron apoptotic process	–
3	IKBKE	16676592	92.0	164.2	0.84	DNA damage response, signal transduction resulting in induction of apoptosis	Apoptosis_KEGG
4	PRKCA	16837128	270.8	456.4	0.75	apoptotic process//cell adhesion//induction of apoptosis by extracellular signals//negative regulation of glial cell apoptotic process	G_Protein_Signaling//Wnt_signaling//Calcium_regulation_in_cardiac_cells//Smooth_muscle_contraction
5	RASGRF2	16997688	76.4	128.0	0.74	apoptotic process	–
6	BIRC3	16730522	36.5	61.0	0.74	apoptotic process//anti-apoptosis	–
7	TAX1BP1	17044568	644.6	1059.6	0.72	apoptotic process//anti-apoptosis	–
8	WT1	16737105	55.6	87.2	0.65	induction of apoptosis//negative regulation of apoptotic process	–
9	FAIM	16946207	103.5	160.1	0.63	apoptotic process//anti-apoptosis	–
10	CAV1	17050578	229.5	354.3	0.63	induction of apoptosis by extracellular signals//positive regulation of anti-apoptosis//positive regulation of extrinsic apoptotic signaling pathway//positive regulation of intrinsic apoptotic signaling pathway	Integrin-mediated_cell_adhesion_KEGG
11	API5	16737482	60.1	92.2	0.62	apoptotic process//anti-apoptosis	–
12	AKAP13	16812871	88.4	135.5	0.62	apoptotic process//induction of apoptosis by extracellular signals	–
13	STK17B	16906733	92.1	139.3	0.60	apoptotic process//induction of apoptosis	–
14	CASP6	16978959	136.6	204.5	0.58	apoptotic process//induction of apoptosis//cellular component disassembly involved in apoptotic process	–
15	MLH1	16938899	469.8	700.4	0.58	DNA damage response, signal transduction resulting in induction of apoptosis	Ovarian_Infertility_Genes

When these genes were assessed by DAVID under the statistical condition of *p* < 0.05, we found 17 terms in the GO-BP, of which the top five were ‘intrinsic apoptotic signaling pathway in response to DNA damage,’ ‘negative regulation of apoptotic process,’ ‘apoptotic process,’ ‘protein homooligomerization,’ and ‘negative regulation of necroptotic process’ (Table 2b). We also found three terms in the KEGG pathway (Table 2b). Of the total GO-BP terms, eight were related to apoptosis, while one was related with anti-apoptosis (Table 2b). In the KEGG pathway, hsa04210, three detected genes, *BIRC3*(*IAPXIP* in KEGG), *BID,* and *CASP6*, exhibited expression differences in SCK-cryopreserved hiPS cells higher than those in non-frozen hiPS cells. Because *BIRC3* suppressed *CASP3*, *CASP7,* and *CAPS9*, which inhibit changes of substrates, it likely caused weakened apoptosis. *BIRC3* is a pro-survival gene itself, and therefore suppresses overall apoptosis (Fig. S1). Furthermore, GeneMANIA analysis of the 15 genes showed that they were strongly expressed and formed one large gene network of physical interaction and pathways (Fig. 2b, Fig. S1).

**Table 2b:** DAVID analysis for genes of SCK-cryopreserved hiPS cells classified under ‘apoptosis’ with a difference in expression of more than 1.5-fold compared with non-frozen hiPS cells (p < 0.05).

No.	Category	Term	Count	%	PValue	Genes	List Total	Pop Hits	Pop Total	Fold Enrichment	Bonferroni	Benjamini	FDR
1	GOTERM_BP_DIRECT	GO:0008630∼intrinsic apoptotic signaling pathway in response to DNA damage	3	23.1	3.40E-04	IKBKE BCL2A1 MLH1	11	47	16792	97.44	0.079	0.083	0.082
2	GOTERM_BP_DIRECT	GO:0043066∼negative regulation of apoptotic process	4	30.8	0.002	BIRC3 FAIM BCL2A1 WT1	11	455	16792	13.42	0.394	0.231	0.229
3	GOTERM_BP_DIRECT	GO:0006915∼apoptotic process	4	30.8	0.004	BIRC3 FAIM CASP6 STK17B	11	567	16792	10.77	0.608	0.231	0.229
4	GOTERM_BP_DIRECT	GO:0051260∼protein homooligomerization	3	23.1	0.005	IKBKE CAV1 BID	11	177	16792	25.87	0.682	0.231	0.229
5	GOTERM_BP_DIRECT	GO:0060546∼negative regulation of necroptotic process	2	15.4	0.005	BIRC3 CAV1	11	8	16792	381.64	0.686	0.231	0.229
6	GOTERM_BP_DIRECT	GO:0042981∼regulation of apoptotic process	3	23.1	0.007	BIRC3 BID CASP6	11	213	16792	21.50	0.807	0.273	0.271
7	GOTERM_BP_DIRECT	GO:0043065∼positive regulation of apoptotic process	3	23.1	0.013	BCL2A1 BID WT1	11	300	16792	15.27	0.959	0.402	0.399
8	GOTERM_BP_DIRECT	GO:0001836∼release of cytochrome *c* from mitochondria	2	15.4	0.014	BCL2A1 BID	11	23	16792	132.74	0.964	0.402	0.399
9	GOTERM_BP_DIRECT	GO:2001238∼positive regulation of extrinsic apoptotic signaling pathway	2	15.4	0.015	CAV1 BID	11	26	16792	117.43	0.977	0.402	0.399
10	GOTERM_BP_DIRECT	GO:0035666∼TRIF-dependent toll-like receptor signaling pathway	2	15.4	0.017	IKBKE BIRC3	11	28	16792	109.04	0.983	0.402	0.399
11	GOTERM_BP_DIRECT	GO:2001244∼positive regulation of intrinsic apoptotic signaling pathway	2	15.4	0.019	CAV1 BID	11	33	16792	92.52	0.992	0.430	0.427
12	GOTERM_BP_DIRECT	GO:0006468∼protein phosphorylation	3	23.1	0.029	IKBKE PRKCA STK17B	11	456	16792	10.04	0.999	0.580	0.576
13	GOTERM_BP_DIRECT	GO:0001570∼vasculogenesis	2	15.4	0.033	CAV1 WT1	11	56	16792	54.52	1.000	0.593	0.588
14	GOTERM_BP_DIRECT	GO:0031398∼positive regulation of protein ubiquitination	2	15.4	0.037	BIRC3 CAV1	11	64	16792	47.70	1.000	0.593	0.588
15	GOTERM_BP_DIRECT	GO:0038061∼NIK/NF-kappaB signaling	2	15.4	0.039	IKBKE BIRC3	11	66	16792	46.26	1.000	0.593	0.588
16	GOTERM_BP_DIRECT	GO:0030855∼epithelial cell differentiation	2	15.4	0.041	WT1 CASP6	11	70	16792	43.62	1.000	0.593	0.588
17	GOTERM_BP_DIRECT	GO:0097190∼apoptotic signaling pathway	2	15.4	0.041	PRKCA CAV1	11	71	16792	43.00	1.000	0.593	0.588
18	KEGG_PATHWAY	hsa04210:Apoptosis	3	23.08	0.002	BIRC3 BID CASP6	9	62	6879	36.98	0.163	0.177	0.177
19	KEGG_PATHWAY	hsa05200:Pathways in cancer	4	30.77	0.008	BIRC3 PRKCA BID MLH1	9	393	6879	7.78	0.497	0.342	0.342
20	KEGG_PATHWAY	hsa04510:Focal adhesion	3	23.08	0.022	BIRC3 PRKCA CAV1	9	206	6879	11.13	0.841	0.606	0.606

Next, we extracted 13 genes that demonstrated a difference in expression of more than 1.5-fold between DAP-cryopreserved and non-frozen hiPS cells. Four of these genes, *BCL2A1*, *CD28*, *NME5*, and *IL10*, were related to anti-apoptosis, while the remaining nine, *BID*, *PRKCA*, *STK17B*, *MLH1*, *PTH*, *C9*, *HIPK2*, *BMF*, and *LALBA*, were apoptosis-related (Table 3a). When we analyzed these genes by DAVID under the statistical condition of *p* < 0.05, we found ten terms in GO-BP, of which the top three were ‘GO:0032464∼positive regulation of protein homooligomerization,’ ‘GO:0043065∼positive regulation of apoptotic process,’ and ‘GO:0001836∼release of cytochrome *c* from mitochondria’ (Table3a, Table 3ba, b). Additionally, four terms in the KEGG pathway that were unrelated to apoptosis were noted (Table3a, Table 3ba, b). The GeneMANIA analysis of these genes revealed a network composed of ten genes showing physical interaction and pathways. However, the expression of these genes was weak, and the network was not as strong as in the SCK-cryopreserved hiPS cells (Fig. 2c). The common apoptotic genes observed in the SCK- and DAP-cryopreserved hiPS cells with a difference in expression greater than 1.5-fold were *BCL2A1*, *BID*, *PRKCA*, *STK17B*, and *MLH*, while the other ten genes for SCK- and eight genes for DAP-cryopreserved hiPS cells were in independent groups (Fig. 2d).

**Table3a:** Genes of DAP-cryopreserved hiPS cells classified under ‘apoptosis’ with a difference in expression of more than 1.5-fold compared with non-frozen hiPS cells.

No	Gene Symbol	Probe Set ID	control2_Signal	DAP_Signal	DAP_Signal Log Ratio	GO Biological Process Term	Pathway Name
1	PTH	16735970	5.3	14.4	1.43	induction of apoptosis by hormones	–
2	C9	16995629	17.7	36.9	1.06	induction of apoptosis	Complement_Activation_Classical
3	BCL2A1	16812344	40.4	80.0	0.99	apoptotic process//anti-apoptosis	Apoptosis_KEGG
4	PRKCA	16837128	270.8	451.2	0.74	induction of apoptosis by extracellular signals	G_Protein_Signaling//Wnt_signaling//Calcium_regulation_in_cardiac_cells//Smooth_muscle_contraction
5	CD28	16889807	24.4	40.4	0.72	induction of apoptosis by extracellular signals//positive regulation of anti-apoptosis	Inflammatory_Response_Pathway
6	STK17B	16906733	92.1	147.7	0.68	apoptotic process//induction of apoptosis	–
7	HIPK2	17063461	676.2	1073.1	0.67	apoptotic process//induction of apoptosis by intracellular signals//DNA damage response, signal transduction by p53 class mediator resulting in induction of apoptosis//negative regulation of neuron apoptotic process	–
8	MLH1	16938899	469.8	741.7	0.66	DNA damage response, signal transduction resulting in induction of apoptosis	Ovarian_Infertility_Genes
9	BMF	16807324	95.1	148.4	0.64	apoptotic process//induction of apoptosis by intracellular signals//activation of pro-apoptotic gene products	–
10	LALBA	16763931	26.9	41.6	0.63	induction of apoptosis	–
11	BID	16932008	309.5	469.2	0.60	apoptotic process//induction of apoptosis by intracellular signals//activation of pro-apoptotic gene products//apoptotic mitochondrial changes//glial cell apoptotic process//regulation of cell proliferation//positive regulation of apoptotic process//neuron apoptotic process//positive regulation of extrinsic apoptotic signaling pathway	–
12	NME5	17000342	22.9	34.4	0.59	anti-apoptosis	–
13	IL10	16698684	22.2	33.1	0.58	anti-apoptosis	–

**Table 3b:** DAVID analysis for genes of DAP-cryopreserved hiPS cells classified under ‘apoptosis’ with a difference in expression of more than 1.5-fold compared with the non-frozen hiPS cells (p < 0.05).

No.	Category	Term	Count	%	PValue	Genes	List Total	Pop Hits	Pop Total	Fold Enrichment	Bonferroni	Benjamini	FDR
1	GOTERM_BP_DIRECT	GO:0032464∼positive regulation of protein homooligomerization	2	15.38	0.005	BID BMF	11	9	16792	339.23	0.722	0.665	0.665
2	GOTERM_BP_DIRECT	GO:0043065∼positive regulation of apoptotic process	3	23.08	0.013	BCL2A1 BID BMF	11	300	16792	15.27	0.956	0.665	0.665
3	GOTERM_BP_DIRECT	GO:0001836∼release of cytochrome *c* from mitochondria	2	15.38	0.014	BCL2A1 BID	11	23	16792	132.74	0.962	0.665	0.665
4	GOTERM_BP_DIRECT	GO:0090200∼positive regulation of release of cytochrome *c* from mitochondria	2	15.38	0.017	BID BMF	11	28	16792	109.04	0.981	0.665	0.665
5	GOTERM_BP_DIRECT	GO:0045944∼positive regulation of transcription from RNA polymerase II promoter	4	30.77	0.018	CD28 IL10 PTH HIPK2	11	981	16792	6.22	0.985	0.665	0.665
6	GOTERM_BP_DIRECT	GO:1900740∼positive regulation of protein insertion into mitochondrial membrane involved in apoptotic signaling pathway	2	15.38	0.018	BID BMF	11	30	16792	101.77	0.986	0.665	0.665
7	GOTERM_BP_DIRECT	GO:2001244∼positive regulation of intrinsic apoptotic signaling pathway	2	15.38	0.019	BID BMF	11	33	16792	92.52	0.991	0.665	0.665
8	GOTERM_BP_DIRECT	GO:0008630∼intrinsic apoptotic signaling pathway in response to DNA damage	2	15.38	0.028	BCL2A1 MLH1	11	47	16792	64.96	0.999	0.761	0.761
9	GOTERM_BP_DIRECT	GO:0006468∼protein phosphorylation	3	23.08	0.029	PRKCA HIPK2 STK17B	11	456	16792	10.04	0.999	0.761	0.761
10	GOTERM_BP_DIRECT	GO:0097190∼apoptotic signaling pathway	2	15.38	0.041	CD28 PRKCA	11	71	16792	43.00	1.000	0.992	0.992
11	KEGG_PATHWAY	hsa05143:African trypanosomiasis	2	15.38	0.033	IL10 PRKCA	8	33	6879	52.11	0.958	0.887	0.887
12	KEGG_PATHWAY	hsa05330:Allograft rejection	2	15.38	0.037	CD28 IL10	8	37	6879	46.48	0.971	0.887	0.887
13	KEGG_PATHWAY	hsa04672:Intestinal immune network for IgA production	2	15.38	0.047	CD28 IL10	8	47	6879	36.59	0.989	0.887	0.887
14	KEGG_PATHWAY	hsa05320:Autoimmune thyroid disease	2	15.38	0.052	CD28 IL10	8	52	6879	33.07	0.993	0.887	0.887

**Fig. 3:**
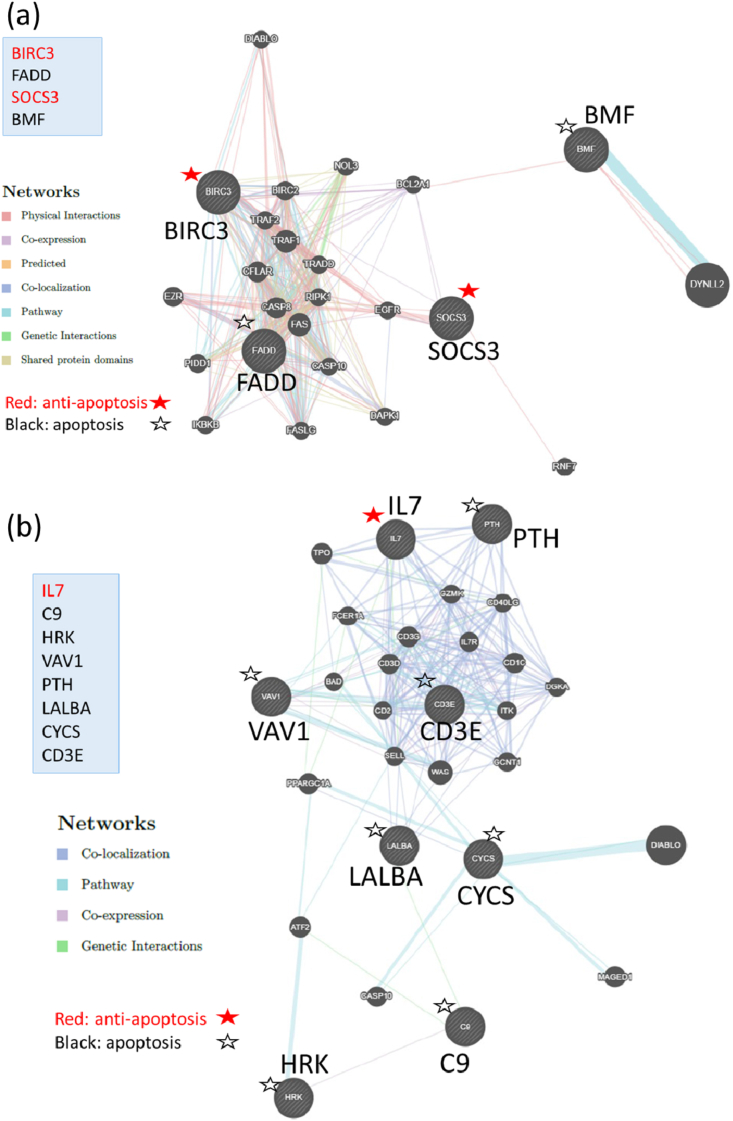
Annotation analysis profiles of SCK- and DAP- cryopreserved hiPS cells (II). (a) GeneMANIA profile of genes of SCK-cryopreserved hiPS cells classified under ‘apoptosis’ with a difference in expression of more than 1.4-fold compared with the DAP-cryopreserved hiPS cells. (b) GeneMANIA profile of genes of DAP-cryopreserved hiPS cells classified under ‘apoptosis’ with a difference in expression of more than 1.4-fold compared with the SCK-cryopreserved hiPS cells. Red font indicates anti-apoptotic genes, and black indicates apoptotic genes. (For interpretation of the references to color in this figure legend, the reader is referred to the Web version of this article.)

*BIRC3*, *FADD*, *SOCS3*, and *BMF3* were expressed by SCK-cryopreserved hiPS cells and demonstrated a difference in expression of over 1.4-fold compared with the DAP-cryopreserved hiPS cells. Among these genes, *BIRC3* and *SOCS3* were connected to anti-apoptosis functions, while *FADD* and *BMF3* connected to apoptosis. (Table 4a). The GeneMANIA analysis showed that *BIRC3*, *FADD*, and *SOCS3* formed strong gene networks with physical interactions, co-expression, and pathways (Fig. 3a).

In contrast, *IL7*, *PTH*, *VAV1*, *LALBA*, *CD3E*, *HRK*, *CYCS*, and *C9* were expressed by DAP-cryopreserved hiPS cells and a difference in expression of over 1.4-fold compared with the SCK-cryopreserved hiPS cells. Among these genes, only *IL7* was associated with an anti-apoptotic function. We further analyzed these genes by DAVID, and extracted five terms in GO-BP and two terms in the KEGG pathway (Table 4a, Table 4b, Table 4ca–c). The GeneMANIA analysis demonstrated that four of these genes, *IL7*, *PTH*, *VAV1*, and *CD3E*, formed a strong gene network with co-localization, co-expression, and pathways (Fig. 3b). Thus, our data indicated that the genes classified under ‘apoptosis’ in SCK-cryopreserved hiPS cells formed a strong gene network with both apoptotic and anti-apoptotic functions.

**Table 4a:** Genes of SCK-cryopreserved hiPS cells classified under ‘apoptosis’ with a difference in expression of more than 1.4-fold when comparing with DAP-cryopreserved hiPS cells.

No.	Gene Symbol	Probe Set ID	control2_Signal	SCK_Signal	SCK_Signal Log Ratio	DAP_Signal	DAP_Signal Log Ratio	Difference	GO Biological Process Term	Pathway Name
1	BIRC3	16730522	36.5	61.0	0.739	29.7	−0.30	1.04	apoptotic process//anti-apoptosis	
2	FADD	16741426	219.9	277.6	0.336	187.9	−0.23	0.56	apoptotic process//activation of cysteine-type endopeptidase activity involved in apoptotic process//induction of apoptosis by extracellular signals//induction of apoptosis via death domain receptors//activation of pro-apoptotic gene products//positive regulation of apoptotic process//extrinsic apoptotic signaling pathway	Apoptosis//Apoptosis_GenMAPP//Apoptosis_KEGG
3	SOCS3	17117736	72.1	84.4	0.228	58.2	−0.31	0.54	anti-apoptosis	
4	BMF	16799423	24.9	35.9	0.527	25.0	0.01	0.52	apoptotic process//induction of apoptosis by intracellular signals//activation of pro-apoptotic gene products ]

**Table 4b:** Genes of DAP-cryopreserved hiPS cells classified under ‘apoptosis’ with a difference in expression of more than 1.4-fold, when comparing between SCK-cryopreserved and DAP-cryopreserved hiPS cells.

No.	Gene Symbol	Probe Set ID	control2_Signal	SCK_Signal	SCK_Signal Log Ratio	DAP_Signal	DAP_Signal Log Ratio	Difference	GO Biological Process Term	Pathway Name
1	PTH	16735970	5.3	7.5	0.49	14.4	1.43	0.94	induction of apoptosis by hormones	–
2	IL7	17078434	23.8	17.2	−0.47	29.6	0.31	0.78	anti-apoptosis	–
3	VAV1	16857490	87.9	61.2	−0.52	98.8	0.17	0.69	apoptotic process	–
4	LALBA	16763931	26.9	27.2	0.02	41.6	0.63	0.61	induction of apoptosis	–
5	CD3E	16731795	36.9	33.6	−0.14	49.3	0.42	0.55	induction of apoptosis by extracellular signals//regulation of apoptotic process	–
6	HRK	16770799	70.3	42.1	−0.74	61.4	−0.20	0.54	apoptotic process//induction of apoptosis//positive regulation of apoptotic process//positive regulation of neuron apoptotic process	–
7	CYCS	17055970	159.6	90.6	−0.82	131.1	−0.28	0.53	apoptotic DNA fragmentation//apoptotic process//induction of apoptosis by intracellular signals//activation of cysteine-type endopeptidase activity involved in apoptotic process by cytochrome *c*	Apoptosis//Apoptosis_GenMAPP//Apoptosis_KEGG
8	C9	16995629	17.7	26.0	0.56	36.9	1.06	0.50	induction of apoptosis//activation of cysteine-type endopeptidase activity involved in apoptotic process	Complement_Activation_Classical

**Table 4c:** DAVID analysis for genes of DAP-cryopreserved hiPS cells classified under “apoptosis” with a difference in expression of more than 1.4-fold, when compared with SCK-cryopreserved hiPS cells (p < 0.05).

No	Category	Term	Count	%	PValue	Genes	List Total	Pop Hits	Pop Total	Fold Enrichment	Bonferroni	Benjamini	FDR
1	GOTERM_BP_DIRECT	GO:0045453∼bone resorption	2	28.6	0.005	PTH IL7	5	22	16792	305.3	0.363	0.296	0.296
2	GOTERM_BP_DIRECT	GO:0048873∼homeostasis of number of cells within a tissue	2	28.6	0.007	PTH IL7	5	29	16792	231.6	0.448	0.296	0.296
3	GOTERM_BP_DIRECT	GO:0007186∼G-protein coupled receptor signaling pathway	3	42.9	0.02	PTH CD3E VAV1	5	899	16792	11.2	0.75	0.397	0.397
4	GOTERM_BP_DIRECT	GO:0031295∼T cell costimulation	2	28.6	0.02	CD3E VAV1	5	78	16792	86.1	0.798	0.397	0.397
5	GOTERM_BP_DIRECT	GO:0010468∼regulation of gene expression	2	28.6	0.02	PTH IL7	5	100	16792	67.2	0.872	0.406	0.406
6	KEGG_PATHWAY	hsa04640:Hematopoietic cell lineage	2	28.6	0.03	IL7 CD3E	3	87	6879	52.7	0.368	0.26	0.26
7	KEGG_PATHWAY	hsa04660:T cell receptor signaling pathway	2	28.6	0.03	CD3E VAV1	3	100	6879	45.9	0.41	0.26	0.26

### 3.3 Enrichment analysis of ‘cell adhesion,’ ‘cell proliferation,’ and ‘stem cell’ in SCK-cryopreserved hiPS cells

During the revival and subsequent culture period, cryopreserved hiPS cells are exposed to environmental stress, and must maintain sufficient proliferation, adhesion, and stemness. Hence, we selected three keywords, ‘cell adhesion,’ ‘cell proliferation,’ and ‘stem cell’ in GO-BP from the DNA microarray data and examined the genes of SCK-cryopreserved hiPS cells that exhibited a difference in expression of over 1.4-fold compared with the DAP-cryopreserved hiPS cells (Table 1). With respect to ‘cell adhesion,’ 11 genes (*ENG*, *PCDH11X*, *PCDHB8*, *PVRL1*, *HAPLN1*, *TNFAIP6*, *WNT5A*, *VCAN*, *CCL4*, *CD209*, and *SELL*) were extracted. Apart from *VCAN* and *CD209*, the differences in gene expression in the DAP-cryopreserved hiPS cells were lower than those in the non-frozen hiPS cells. Additionally, the number of SCK-cryopreserved hiPS cells that survived post revival was higher than that of DAP-cryopreserved hiPS cells. Furthermore, genes such as *HAPLN1*, *TNFAIP6*, and *CCL4* of DAP-cryopreserved hiPS cells were expressed at lower levels than those of in non-frozen cells.

**Fig. 4:**
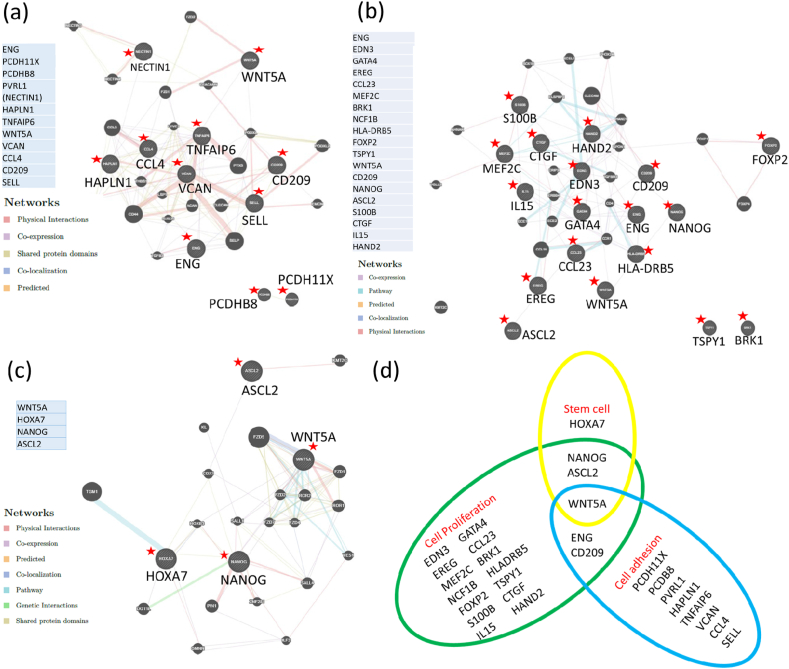
Annotation analysis profiles of SCK- or DAP- cryopreserved hiPS cells (III). (a) GeneMANIA profile of genes of SCK-cryopreserved hiPS cells classified under ‘cell adhesion’ that exhibited a difference in expression larger than that of DAP-cryopreserved hiPS cells. (b) GeneMANIA profile of genes of SCK-cryopreserved hiPS cells classified under ‘cell proliferation’ with a difference in expression of more than 1.4-fold compared with the DAP-cryopreserved hiPS cells. (c) GeneMANIA profile of genes of SCK-cryopreserved hiPS cells classified under ‘stem cell’ with a difference in expression of more than 1.4-fold compared with the DAP-cryopreserved hiPS cells. Red stars indicate selected genes in a, b and c. (d) Gene expression profile of SCK-cryopreserved hiPS cells that exhibited a difference in expression of more than 1.4-fold compared with the DAP-cryopreserved hiPS cells. (For interpretation of the references to color in this figure legend, the reader is referred to the Web version of this article.)

The average signal log ratio of 11 genes in the SCK-cryopreserved hiPS cells was 0.28 ± 0.37, and was significantly higher than that of DAP-cryopreserved hiPS cells (−0.37 ± 0.36; *p* = 4.1 × 10^-8^; Table 5a). When we analyzed these genes by DAVID, we found five terms in GO-BP, of which the top three were ‘GO:0007155∼cell adhesion,’ ‘GO:0007157∼heterophilic cell-cell adhesion via plasma membrane cell adhesion molecules,’ and ‘GO:0071346∼cellular response to interferon-gamma’ (Table 5b). Additionally, the GeneMANIA analysis demonstrated that, although the gene expression was not strong, there were gene networks formed between *HAPLN1*, *TNFAIP6*, *VCAN*, *CCL4*, *CD209*, and *SELL* with co-expression and pathways (Fig. 4a).

**Table 5a:** Genes of SCK-cryopreserved hiPS cells classified under ‘cell adhesion’ with a difference in expression of more than 1.4-fold when compared with DAP-cryopreserved hiPS cells.

No.	Gene Symbol	Probe Set ID	control2_Signal	SCK_Signal	SCK_Signal Log Ratio	DAP_Signal	DAP_Signal Log Ratio	Difference	GO Biological Process Term
1	ENG	17098594	255.7	380.6	0.57	193.7	−0.4	0.97	cell adhesion
2	PCDH11X	17105249	198.4	279.3	0.49	144.2	−0.46	0.95	homophilic cell adhesion
3	PCDHB8	16990284	30.3	33.1	0.13	21	−0.53	0.66	homophilic cell adhesion
4	PVRL1 (NECTIN1)	16745380	112	128.5	0.2	83	−0.43	0.63	cell adhesion//homophilic cell adhesion//heterophilic cell-cell adhesion//cell-cell adhesion//adherens junction organization
5	HAPLN1	16997802	75.2	73.8	−0.03	48.6	−0.63	0.6	cell adhesion
6	TNFAIP6	16886491	584.6	458	−0.35	305.2	−0.94	0.59	cell adhesion
7	WNT5A	16955197	118.4	119.6	0.01	80	−0.57	0.58	positive regulation of cell-cell adhesion mediated by cadherin
8	VCAN	16997799	102.6	202.2	0.98	136.9	0.42	0.56	cell adhesion
9	CCL4	16833420	41.4	40.8	−0.02	27.8	−0.57	0.55	cell adhesion
10	CD209	16868000	95.9	149.2	0.64	102	0.09	0.55	heterophilic cell-cell adhesion//leukocyte cell-cell adhesion
11	SELL	16696237	34.9	49.5	0.5	34.1	−0.03	0.54	cell adhesion

**Table 5b:** DAVID analysis for genes of SCK-cryopreserved hiPS cells classified under ‘cell adhesion’ with a difference in expression of more than 1.4-fold when compared with DAP-cryopreserved hiPS cells (p < 0.05).

No.	Category	Term	Count	%	PValue	Genes	List Total	Pop Hits	Pop Total	Fold Enrichment	Bonferroni	Benjamini	FDR
1	GOTERM_BP_DIRECT	GO:0007155∼cell adhesion	4	40	3.82E-04	CCL4 PVRL1 SELL TNFAIP6	7	459	16792	20.91	0.068	0.07	0.07
2	GOTERM_BP_DIRECT	GO:0007157∼heterophilic cell-cell adhesion via plasma membrane cell adhesion molecules	2	20	0.02	PVRL CD209	7	50	16792	95.95	0.963	1	1
3	GOTERM_BP_DIRECT	GO:0071346∼cellular response to interferon-gamma	2	20	0.02	CCL4 WNT5A	7	57	16792	84.17	0.977	1	1
4	GOTERM_BP_DIRECT	GO:0050729∼positive regulation of inflammatory response	2	20	0.03	CCL4 WNT5A	7	73	16792	65.72	0.992	1	1
5	GOTERM_BP_DIRECT	GO:0046718∼viral entry into host cell	2	20	0.03	PVRL CD209	7	80	16792	59.97	0.995	1	1
6	INTERPRO	IPR016186:C-type lectin-like	3	30	4.60E-04	CD209 SELL TNFAIP6	7	104	18559	76.48	0.012	0.007	0.007
7	INTERPRO	IPR016187:C-type lectin fold	3	30	5.33E-04	CD209 SELL TNFAIP6	7	112	18559	71.02	0.014	0.007	0.007
8	INTERPRO	IPR018378:C-type lectin, conserved site	2	20	0.01	CD209 SELL	7	44	18559	120.51	0.309	0.123	0.123
9	INTERPRO	IPR001304:C-type lectin	2	20	0.03	CD209 SELL	7	89	18559	59.58	0.528	0.185	0.185
10	SMART	SM00034:CLECT	2	20	0.05	CD209 SELL	7	86	10057	33.41	0.433	0.553	0.553

With respect to ‘cell proliferation,’ 19 genes (*ENG, EDN3, GATA4, EREG, CCL23, MEF2C, BRK1, NCF1B, HLS-DRB5, FOXP2, TSPY1, WNT5A, CD209, NANOG, ASCL2, S100B, CTGF, IL15,* and *HAND2*) of SCK-cryopreserved hiPS cells were found to exhibit a difference in expression of over 1.4-fold compared with the DAP-cryopreserved hiPS cells. Notably, the signal log ratios of DAP-cryopreserved hiPS cells were found to be negative in all but three genes: *BRK1, CD209,* and *S100B*. In contrast, the differences in gene expression of SCK-cryopreserved hiPS cells were mostly positive, except for three genes: *FOXP2, ASCL2,* and *IL15*. The average signal log ratio of the 19 genes of SCK-cryopreserved hiPS cells was 0.30 ± 0.31, which was significantly higher than that of DAP-cryopreserved hiPS cells (−0.36 ± 0.27; *p* = 1.3 × 10^-14^; Table 6a). When we assessed these genes by DAVID, we found 17 terms in GO-BP, of which the top three were ‘GO:0007267∼cell-cell signaling,’ ‘GO:0008284∼positive regulation of cell proliferation,’ and ‘GO:0001947∼heart looping,’ and two terms in the KEGG pathway (Table 6b). The GeneMANIA analysis showed one gene network group made up of 13 genes, apart from *BRK1, FOXP2, TSPY1*, and *ASCL2*, with co-expression and pathways. However, their pathways and physical interactions were found to be weak (Fig. 4b).

**Table 6a:** Genes of SCK-cryopreserved hiPS cells classified under ‘cell proliferation’ with a difference in expression of more than 1.4-fold when compared with DAP-cryopreserved hiPS cells.

No.	Gene Symbol	Probe Set ID	control2_Signal	SCK_Signal	SCK_Signal Log Ratio	DAP_Signal	DAP_Signal Log Ratio	difference	GO Biological Process Term
1	ENG	17098594	255.7	380.6	0.57	193.7	−0.4	0.97	regulation of cell proliferation
2	EDN3	16915412	68.6	115.2	0.75	65.1	−0.08	0.82	positive regulation of cell proliferation
3	GATA4	17065780	50.7	68.8	0.44	39.1	−0.37	0.81	positive regulation of cardiac muscle cell proliferation
4	EREG	16967843	38.4	55.3	0.52	31.7	−0.28	0.8	positive regulation of cell proliferation//negative regulation of cell proliferation//keratinocyte proliferation//positive regulation of smooth muscle cell proliferation//negative regulation of epithelial cell proliferation//negative regulation of smooth muscle cell differentiation
5	CCL23	16843567	36.2	50.7	0.49	29.3	−0.31	0.79	negative regulation of cell proliferation
6	MEF2C	16997953	34.7	41.7	0.27	25.3	−0.46	0.72	positive regulation of B cell proliferation//muscle cell differentiation//positive regulation of cardiac muscle cell proliferation//epithelial cell proliferation involved in renal tubule morphogenesis
7	BRK1	17118001	105.1	177.5	0.76	107.7	0.03	0.72	positive regulation of cell proliferation
8	NCF1B	17046911	24.7	33.3	0.43	20.3	−0.28	0.71	cell proliferation
9	HLA-DRB5	17017885	14.8	15.2	0.04	9.8	−0.59	0.63	negative regulation of T cell proliferation
10	FOXP2	17050455	46.8	41.6	−0.17	27	−0.79	0.62	positive regulation of mesenchymal cell proliferation//positive regulation of epithelial cell proliferation involved in lung morphogenesis
11	TSPY1	17116131	50.4	60.9	0.27	39.6	−0.35	0.62	cell proliferation
12	WNT5A	16955197	118.4	119.6	0.01	80	−0.57	0.58	positive regulation of endothelial cell proliferation//positive regulation of mesenchymal cell proliferation//epithelial cell proliferation involved in mammary gland duct elongation//hemopoietic stem cell proliferation//negative regulation of mesenchymal cell proliferation
13	CD209	16868000	95.9	149.2	0.64	102	0.09	0.55	regulation of T cell proliferation
14	NANOG	16747852	481.4	507.4	0.08	349	−0.46	0.54	cell proliferation
15	ASCL2	16734420	95.7	74.9	−0.35	51.7	−0.89	0.54	negative regulation of Schwann cell proliferation
16	S100B	16926754	52.7	82.1	0.64	56.7	0.11	0.54	cell proliferation
17	CTGF	17118180	476.1	616.9	0.37	425.8	−0.16	0.53	positive regulation of cell proliferation
18	IL15	16970971	41	40	−0.04	27.8	−0.56	0.52	NK T cell proliferation//positive regulation of cell proliferation//positive regulation of NK cell proliferation//positive regulation of T cell proliferation//negative regulation of smooth muscle cell proliferation
19	HAND2	16981542	86.1	90.6	0.07	63.3	−0.44	0.52	regulation of secondary heart field cardioblast proliferation//mesenchymal cell proliferation

**Table 6b:** DAVID analysis for genes of SCK-cryopreserved hiPS cells classified under ‘cell proliferation’ with a difference in expression of more than 1.4-fold when compared with DAP-cryopreserved hiPS cells (p < 0.05).

No.	Category	Term	Count	%	PValue	Genes	List Total	Pop Hits	Pop Total	Fold Enrichment	Bonferroni	Benjamini	FDR
1	GOTERM_BP_DIRECT	GO:0007267∼cell-cell signaling	5	27.8	4.54E-05	IL15 EREG GATA CCL23 EDN3	15	254	16792	22.04	0.016	0.017	0.017
2	GOTERM_BP_DIRECT	GO:0008284∼positive regulation of cell proliferation	5	27.8	4.70E-04	S100B IL15 EREG EDN3 BRK1	15	466	16792	12.01	0.158	0.086	0.086
3	GOTERM_BP_DIRECT	GO:0001947∼heart looping	3	16.7	0.001	WNT5A HAND2 GATA4	15	61	16792	55.06	0.343	0.14	0.14
4	GOTERM_BP_DIRECT	GO:0050729∼positive regulation of inflammatory response	3	16.7	0.002	WNT5A IL15 CCL23	15	73	16792	46.01	0.452	0.15	0.15
5	GOTERM_BP_DIRECT	GO:0045840∼positive regulation of mitotic nuclear division	2	11.1	0.021	EREG EDN3	15	26	16792	86.11	1	1	1
6	GOTERM_BP_DIRECT	GO:0002053∼positive regulation of mesenchymal cell proliferation	2	11.1	0.021	WNT5A FOXP2	15	26	16792	86.11	1	1	1
7	GOTERM_BP_DIRECT	GO:0008283∼cell proliferation	3	16.7	0.036	S100B TSPY1 NANOG	15	366	16792	9.18	1	1	1
8	GOTERM_BP_DIRECT	GO:0045165∼cell fate commitment	2	11.1	0.038	WNT5A GATA4	15	46	16792	48.67	1	1	1
9	GOTERM_BP_DIRECT	GO:0048146∼positive regulation of fibroblast proliferation	2	11.1	0.044	WNT5A EREG	15	54	16792	41.46	1	1	1
10	GOTERM_BP_DIRECT	GO:0045944∼positive regulation of transcription from RNA polymerase II promoter	4	22.2	0.045	WNT5A HAND2 NANOG GATA4	15	981	16792	4.56	1	1	1
11	GOTERM_BP_DIRECT	GO:0019882∼antigen processing and presentation	2	11.1	0.045	HLA-DRB5 CD209	15	55	16792	40.71	1	1	1
12	GOTERM_BP_DIRECT	GO:0050680∼negative regulation of epithelial cell proliferation	2	11.1	0.046	WNT5A EREG	15	56	16792	39.98	1	1	1
13	GOTERM_BP_DIRECT	GO:0042733∼embryonic digit morphogenesis	2	11.1	0.046	WNT5A HAND2	15	56	16792	39.98	1	1	1
14	GOTERM_BP_DIRECT	GO:0071346∼cellular response to interferon-gamma	2	11.1	0.047	WNT5A CCL23	15	57	16792	39.28	1	1	1
15	GOTERM_BP_DIRECT	GO:0006955∼immune response	3	16.7	0.047	IL15 HLA-DRB5 CCL23	15	421	16792	7.98	1	1	1
16	GOTERM_BP_DIRECT	GO:0035019∼somatic stem cell population maintenance	2	11.1	0.05	ASCL2 NANOG	15	65	16792	34.45	1	1	1
17	GOTERM_BP_DIRECT	GO:0030593∼neutrophil chemotaxis	2	11.1	0.05	CCL23 EDN3	15	66	16792	33.92	1	1	1
18	KEGG_PATHWAY	hsa05166:HTLV-I infection	3	16.7	0.033	WNT5A IL15 HLA-DRB5	9	254	6879	9.03	0.719	0.999	0.999
19	KEGG_PATHWAY	hsa04672:Intestinal immune network for IgA production	2	11.1	0.053	IL15, HLA-DRB5	9	47	6879	32.52	0.876	0.999	0.999

Finally, we extracted four genes of SCK-cryopreserved hiPS cells that presented a difference in expression of over 1.4-fold compared with the DAP-cryopreserved hiPS cells, *WNT5A, HOXA7, NANOG*, and *ASCL2*. The average signal log ratio of these genes in SCK-cryopreserved hiPS cells was -0.03 ± 0.19, which was significantly higher than that of DAP-cryopreserved hiPS cells (−0.58 ± 0.19; *p* = 7.4 × 10^-6^; Table 7a). Finally, when these genes were analyzed by DAVID, we found two terms in the GO-BP: ‘GO:0045944∼positive regulation of transcription from RNA polymerase II promoter’ and ‘GO:0035019∼somatic stem cell population maintenance’ (Table 7a, Table 7ba, b). The GeneMANIA analysis demonstrated a pattern of strong gene expression and gene networks formed by three genes with physical interaction, co-expression, and pathways (Fig. 4c).

**Table 7a:** Genes of SCK-cryopreserved hiPS cells classified under ‘stem cell’ with a difference in expression of more than 1.4-fold when compared with DAP-cryopreserved hiPS cells.

No.	Gene Symbol	Probe Set ID	control2_Signal	SCK_Signal	SCK_Signal Log Ratio	DAP_Signal	DAP_Signal Log Ratio	Difference	GO Biological Process Term
1	WNT5A	16955197	118.4	119.6	0.01	80	−0.57	0.58	hemopoietic stem cell proliferation
2	HOXA7	17056152	91.2	101.2	0.15	69.6	−0.39	0.54	stem cell differentiation
3	NANOG	16747852	481.4	507.4	0.08	349	−0.46	0.54	somatic stem cell maintenance
4	ASCL2	16734420	95.7	74.9	−0.35	51.7	−0.89	0.54	somatic stem cell maintenance

**Table 7b:** DAVID analysis of genes of SCK-cryopreserved hiPS cells classified under ‘stem cell’ with a difference in expression of more than 1.4-fold when compared with DAP-cryopreserved hiPS cells.

No	Category	Term	Count	%	PValue	Genes	List Total	Pop Hits	Pop Total	Fold Enrichment	Bonferroni	Benjamini	FDR
1	GOTERM_BP_DIRECT	GO:0045944∼positive regulation of transcription from RNA polymerase II promoter	3	75	0.01	WNT5A HOXA7 NANOG	4	981	16792	12.84	0.784	0.897	0.897
2	GOTERM_BP_DIRECT	GO:0035019∼somatic stem cell population maintenance	2	50	0.01	ASCL2 NANOG	4	65	16792	129.17	0.835	0.897	0.897
3	KEGG_PATHWAY	hsa04550:Signaling pathways regulating pluripotency of stem cells	2	50	0.02	WNT5A NANOG	2	140	6879	49.14	0.152	0.116	0.116
4	KEGG_PATHWAY	hsa05205:Proteoglycans in cancer	2	50	0.03	WNT5A NANOG	2	200	6879	34.39	0.21	0.116	0.116

The inclusion diagram of genes of SCK-cryopreserved hiPS cells with higher expression than those of DAP-cryopreserved hiPS cells showed that *NANOG, ASCL2, ENG,* and *CD209* were stronger in two functions, while *WNT5A* appeared in three (Fig. 4d). Additionally, the genes *BIRC3*, *BID* and *CASP6*, classified under the keyword ‘apoptosis’ in SCK-cryopreserved hiPS cells and with a difference in expression 1.5-fold greater than that in non-frozen hiPS cells, were also categorized under ‘focal adhesion’ in the KEGG pathway, and were associated with cell-cell adhesion and followed cell survival (Table 2(a) No.1,4,10 and Table 2(b) No.20; Fig. S2).

These findings suggest that SCK-cryopreserved hiPS cells would be more quickly cultured and maintained in good condition after thawing compared to DAP-cryopreserved cells.

## 4 Discussion

There are two cryopreservation methods for stem cells: vitrification and slow freezing [35,36]. Although the freezing volume used for the vitrification of cells is smaller, the damage incurred during freeze-thaw is less than that incurred during slow freezing [[37], [38], [39], [40]]. We previously demonstrated that SCK, a DMSO-free cryopreservation solution, exhibited excellent cryoprotectant properties, especially for the preservation of hiPS cells and hES cells by vitrification. In this study, we reported that SCK-cryopreserved hiPS cells retained their multipotency and pluripotency post-revival [11,12].

After revival, the SCK-cryopreserved hiPS cells were found to proliferate faster and with higher potency when compared with the DAP-cryopreserved hiPS cells (Fig. 1). Notably, when DAP-cryopreserved hiPS cells were thawed and cultured, a large number of cells did not survive the freeze-thaw process. The few surviving cells required a long period of culture in order to generate cells in sufficient numbers to perform an experiment. In contrast, the SCK-cryopreserved hiPS cells exhibited strong adhesive properties to the culture dish and proliferated quickly, even though some cells did not survive the freeze-thaw process. Thus, we concluded that SCK-treated cells were protected from external damage compared with DAP-treated cells. and wanted to identify the genes involved in the effective cryopreservation of SCK-cryopreserved hiPS cells using DNA microarray analysis.

hiPS cells are commonly cryopreserved as cell colonies by vitrification [11,21]. After thawing, the cells are cultured as clots on a feeder layer without making a single cell suspension, but often do not survive this process [34]. Hence, we hypothesized that hiPS cells underwent apoptosis post-revival. In order to address this using DNA microarray data, we first checked the term ‘apoptosis’ in GO-BP, and compared the gene profile of SCK-cryopreserved hiPS cells with those of non-frozen or DAP-cryopreserved hiPS cells with respect to differences in gene expression. We extracted the genes that exhibited a difference in expression of more than 1.5-fold and found 15 genes; seven genes were anti-apoptotic in nature, while the remaining eight genes had an apoptotic function. Hence, both anti-apoptotic and apoptotic genes were competitively expressed in SCK-cryopreserved hiPS cells.

In contrast, we extracted only four anti-apoptotic genes that exhibited a difference in expression of more than 1.5-fold in DAP-cryopreserved hiPS cells versus non-frozen hiPS cells, while the remaining nine genes strongly facilitated apoptosis. Among the apoptotic genes extracted from SCK- and DAP-cryopreserved hiPS cells compared with non-frozen hiPS cells, *BCL2A1, BID, PRIKCA, STK17B* and *MLH1* were commonly expressed, though they fell into in different categories from apoptosis in DAVID analysis. In the hsa04210 apoptosis KEGG pathway, SCK-cryopreserved hiPS cells exhibited three genes, *BIRC3(LAPXIP*), *BID* and *CASP6*, with expression differences higher than those of non-frozen hiPS cells. According to the KEGG pathway, *BID* and *CASP6* enhance apoptosis, whereas *BIRC3* suppresses *CASP3, 7* and *9*, and is a pro-survival gene in itself. This suggests that SCK-cryopreserved hiPS cells would exhibit significantly improved survival after thawing. This speculation needs to be inspected through further experimentation (Table 3a, b; Fig. 2d, Fig. S1).

When we compared the genes between SCK- and DAP-cryopreserved hiPS cells that exhibited a difference in expression of more than 1.5-fold, only one was selected in SCK-cryopreserved hiPS cells. Hence, a less stringent condition (i.e., change of more than 1.4-fold) was applied, allowing four genes (*BIRC3, FADD, SOCS3*, and *BMF*) to be extracted. While there were no effective gene categories extracted in DAVID, however, a strong gene network including *BIRC3, FADD*, and *SOCS3* was formed by physical interaction, co-expression, pathways, and genetic interaction in GeneMANIA. Thus, our data indicated that the anti-apoptotic function of SCK-cryopreserved hiPS cells may be stronger than the apoptotic function.

The three keywords, ‘cell adhesion,’ ‘cell proliferation,’ and ‘stem cell,’ extracted by GO-BP are important factors that play a role in stem cell culture after their revival. We extracted the genes of SCK-cryopreserved hiPS cells in these keywords using a less stringent condition, (genes exhibiting a difference in expression of more than 1.4-fold), as we predicted that the differences between the expression of these genes would not be large. In case of the keyword ‘cell adhesion,’ 11 genes were extracted, of which four fell under the category of ‘cell adhesion’ when analyzed by DAVID. Furthermore, six of the genes exhibited a physical interaction in GeneMANIA. Hence, we concluded that the SCK-cryopreserved hiPS cells demonstrated strong adhesive properties after thawing.

In the case of the keyword, ‘cell proliferation,’ 19 genes were extracted. Out these, 15 were involved in a wide gene network, although their gene expression was not as strong in GeneMANIA. For the keyword ‘stem cell,’ we first used the keyword ‘stem cell maintenance.’ However, as only two genes were selected using this term, we changed the selecting condition to ‘stem cell’. We extracted four genes for this keyword, with two categories in BP-GO and two KEGG pathways found in DAVID analysis. However, no gene network was detected in GeneMANIA regarding the keyword ‘stem cell’. With respect to the gene expression associated with these keywords in SCK- and DAP-cryopreserved hiPS cells, a severe decline in expression was observed in the DAP-cryopreserved hiPS cells, while low to moderate gene expression was observed in the SCK-cryopreserved hiPS cells. Furthermore, these differences in the gene expression profiles could be associated with attachment of the hiPS cells to culture dishes after thawing, cell growth, and maintenance of pluripotency of the stem cells.

Further studies are required to investigate the expression of the individual genes that were reported in this study. Additionally, as only the 253G1 strain of hiPS cells was used in this study, it is important to investigate other hiPS cell strains as well to confirm the reproducibility of this study.

The final goal of this study was to determine the effective genes and their interactions in SCK-cryopreserved hiPS cells. We found that SCK-cryopreserved hiPS cells showed a group of anti-apoptotic genes and other groups related to the keywords ‘cell proliferation,’ ‘cell adhesion,’ and ‘stem cell,’ however, how these genes interact is still unknown. Further studies should research the effects of these genes on cryopreservation through Western blotting, immunochemical staining, reverse transcript PCR, or gene editing methods.

Recently, the development of chemical ice-inhibition molecules, including cryoprotectant, antifreeze protein, synthetic polymer, nanomaterial, and hydrogel, and their applications in regenerative devices and cryopreservation, has progressed. Additionally, advanced engineering strategies, including trehalose delivery, cell encapsulation, and bioinspired structure design for ice inhibition, are also amazingly developed [[41], [42], [43]]. Through the combination of SCK and these novel products or advanced engineering techniques, we expect to improve cryopreservation methods.

In conclusion, the DNA microarray analysis of SCK-cryopreserved hiPS cells demonstrated that apoptotic genes *BID* and *CASP6*, enhanced apoptosis, whereas *BIRC3,* an anti-apoptotic gene, suppressed *CASP3, 7* and *9* in the apoptosis KEGG pathway. Owing to anti-apoptotic function of *BIRC3* as well as genes involved in cell adhesion, cell proliferation, and multipotency, SCK-cryopreserved hiPS cells are likely to exhibit survival and easy culturing after thawing. Thus, SCK is likely superior to DAP for stem cell storage and maintenance. Our results showed that SCK is suitable for the efficient preservation of stem cells that can be used clinically and in basic research for regenerative medicine. While more genetic analysis is needed, we suggest SCK as a superior cryopreservation agent to DAP and more appropriate for clinical use and future investigations.

## Data availability

Data will be made available on request.

## Author disclosure statement

K.M. and S.-H.H. are cofounders of Bioverde Inc.; A.O. is an employee of Bioverde, Inc.

## Declaration of competing interests

The authors declare the following financial interests/personal relationships which may be considered as potential competing interests:
