# Supplementary material for: Gene expression analysis of human induced pluripotent stem cells cryopreserved by vitrification using StemCell Keep
Source: Biochem Biophys Rep. 2021 Nov 15;28:101172. doi: 10.1016/j.bbrep.2021.101172 (PMC8605251; doi:10.1016/j.bbrep.2021.101172)
Supplement: Multimedia component 1 [file mmc1.docx]

Supplementary Data for

**Gene expression analysis of human induced pluripotent stem cells cryopreserved by vitrification using StemCell Keep**

Akemi Ota, Suong-Hyu Hyon, Shoichiro Sumi, Kazuaki Matsumura*


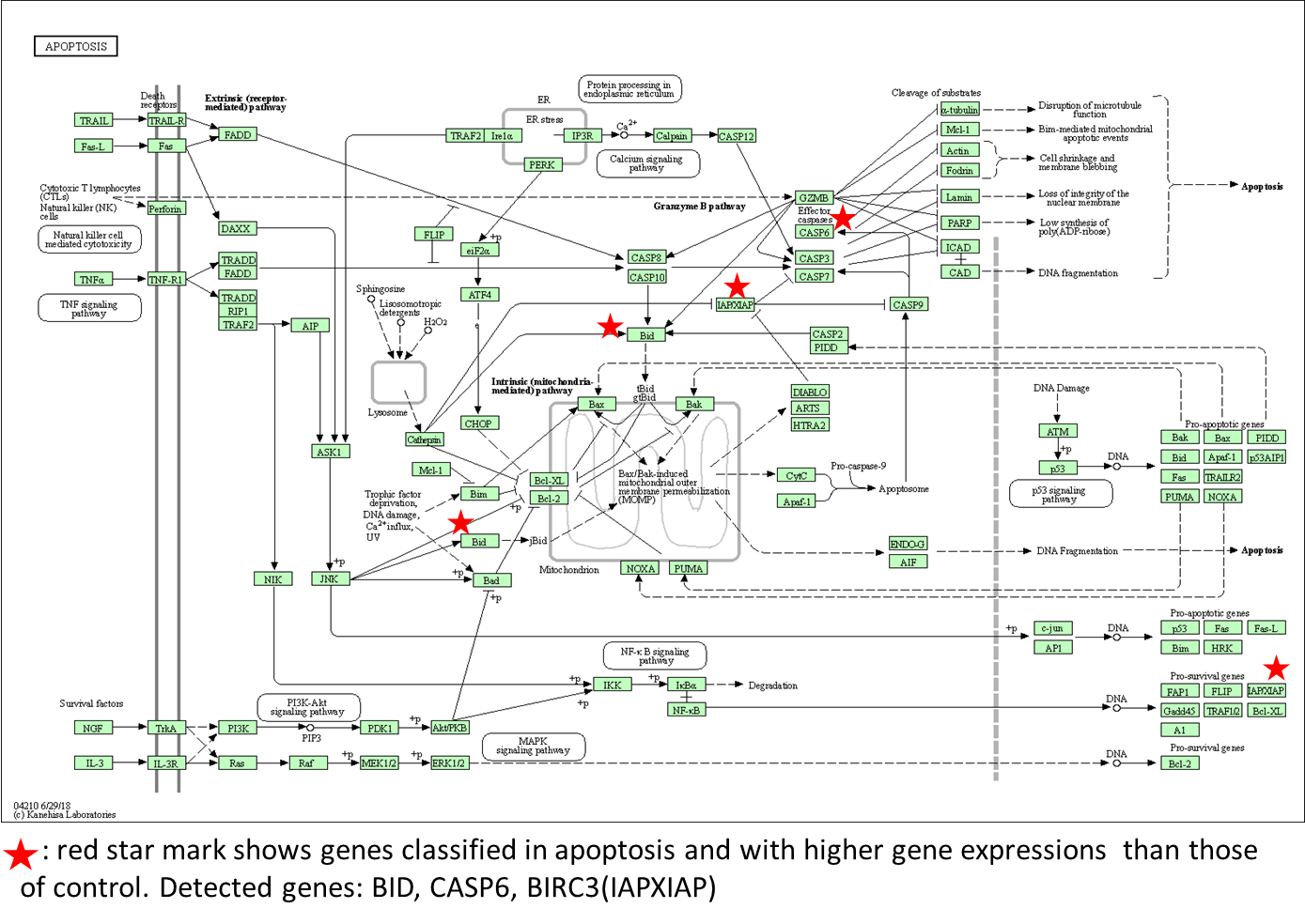


**Figure S1.** The KEGG pathway: hsa04210 showed that *BIRC3*, *BID*, and *CASP6* genes of SCK-cryopreserved hiPS cells demonstrated a large difference in expression compared to the unfrozen hiPS cells.


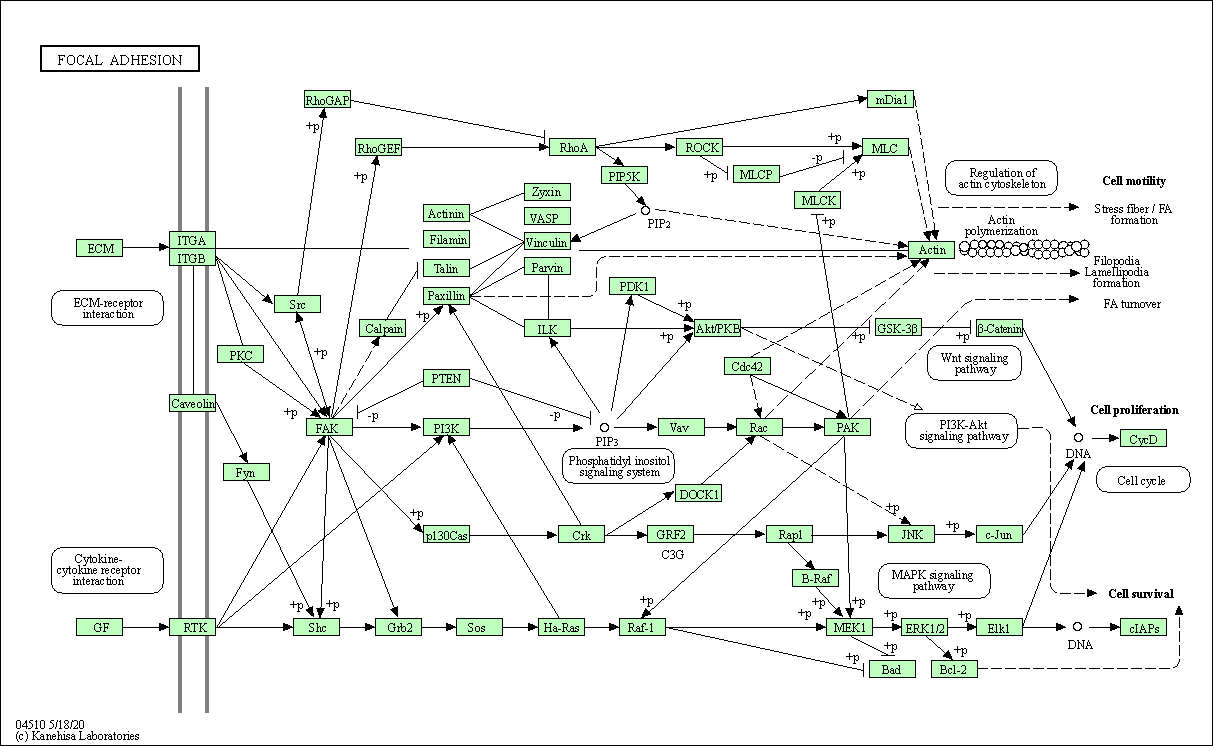


; Genes of selected

**Figure S2.** ‘Focal adhesion’ of KEGG pathways of SCK-cryopreserved hiPS cells classified under ‘apoptosis’ in DAVID analysis.
